# Applications and Limitations of Dendrimers in Biomedicine

**DOI:** 10.3390/molecules25173982

**Published:** 2020-09-01

**Authors:** Adriana Aurelia Chis, Carmen Dobrea, Claudiu Morgovan, Anca Maria Arseniu, Luca Liviu Rus, Anca Butuca, Anca Maria Juncan, Maria Totan, Andreea Loredana Vonica-Tincu, Gabriela Cormos, Andrei Catalin Muntean, Maria Lucia Muresan, Felicia Gabriela Gligor, Adina Frum

**Affiliations:** Preclinical Department, Faculty of Medicine, “Lucian Blaga” University of Sibiu, 2A Lucian Blaga St., 550169 Sibiu, Romania; adriana.chis@ulbsibiu.ro (A.A.C.); anca.arseniu@ulbsibiu.ro (A.M.A.); liviu.rus@ulbsibiu.ro (L.L.R.); anca.butuca@ulbsibiu.ro (A.B.); ancamaria.juncan@ulbsibiu.ro (A.M.J.); maria.totan@ulbsibiu.ro (M.T.); loredana.vonica@ulbsibiu.ro (A.L.V.-T.); gabriela.cormos@ulbsibiu.ro (G.C.); andreicatalin.muntean@ulbsibiu.ro (A.C.M.); maria.muresan@ulbsibiu.ro (M.L.M.); felicia.gligor@ulbsibiu.ro (F.G.G.); adina.frum@ulbsibiu.ro (A.F.)

**Keywords:** cytotoxicity, dendrimers, drug therapy, imagining diagnostics, targeted release

## Abstract

Biomedicine represents one of the main study areas for dendrimers, which have proven to be valuable both in diagnostics and therapy, due to their capacity for improving solubility, absorption, bioavailability and targeted distribution. Molecular cytotoxicity constitutes a limiting characteristic, especially for cationic and higher-generation dendrimers. Antineoplastic research of dendrimers has been widely developed, and several types of poly(amidoamine) and poly(propylene imine) dendrimer complexes with doxorubicin, paclitaxel, imatinib, sunitinib, cisplatin, melphalan and methotrexate have shown an improvement in comparison with the drug molecule alone. The anti-inflammatory therapy focused on dendrimer complexes of ibuprofen, indomethacin, piroxicam, ketoprofen and diflunisal. In the context of the development of antibiotic-resistant bacterial strains, dendrimer complexes of fluoroquinolones, macrolides, beta-lactamines and aminoglycosides have shown promising effects. Regarding antiviral therapy, studies have been performed to develop dendrimer conjugates with tenofovir, maraviroc, zidovudine, oseltamivir and acyclovir, among others. Furthermore, cardiovascular therapy has strongly addressed dendrimers. Employed in imaging diagnostics, dendrimers reduce the dosage required to obtain images, thus improving the efficiency of radioisotopes. Dendrimers are macromolecular structures with multiple advantages that can suffer modifications depending on the chemical nature of the drug that has to be transported. The results obtained so far encourage the pursuit of new studies.

## 1. Introduction

The term “dendrimer” is a combination of two Greek words, “dendron” and “meros”, translated as tree and parts, thus explaining their branched structure [1]. The first idea of branched molecules was stated by Flory in 1941, but the experimental support for it was not enough at that time. The first paper regarding dendritic structure was published by Vögtle and coworkers, in 1978. They created a dendritic structure by using divergent synthesis. Later on, this discovery was confirmed by Denkewalter et al. in 1981, Tomalia et al. in 1983 and Newkome et al. in 1985. The convergent approach was introduced by Hawker and Frechet in 1990 [2].

Dendrimers are synthetic polymers characterized by branched repeating units that emerge from a focal point and possess a large number of exposed anionic, neutral or cationic terminal functionalities on the surface, which leads to hydrophilic or hydrophobic compounds [3]. They are nanometric molecules that are radially symmetric, globular, mono-dispersed and homogenous [4].

The properties of dendrimers are different in comparison to conventional polymers. Due to their size, dendrimers are used in nanomedicine research. They are found to be useful as delivery or carrier systems for drugs and genes, but studies have shown that some dendrimers have medicinal uses of their own, mostly due to their antifungal, antibacterial and cytotoxic properties [5,6].

The benefits of many drugs cannot be exploited because of their poor solubility, toxicity or stability problems. The use of dendrimers as carriers of these compounds can solve these problems, thus improving their clinical applications [7].

The valorization of dendrimers represents an important progress in the current therapeutic field, and the biodegradable properties of these polymers can significantly increase their applicability. Dendrimers’ excretion (hepatic or renal) differs depending on the generation. Moreover, the structural versatility of dendrimers gives them special qualities in the context of using them as ideal carriers for many active drug molecules. In addition, the easy-to-control characteristics of dendrimers (namely: size, shape, liposome blockage in dendrimeric structures, branch length, surface functionality and synthesis of targeted dendritic scaffolds) makes these systems ideal carriers in many applications. The controllable and adjustable size, the interaction with cell membranes and various active drug molecules and the characteristics of their internal structures and cavities, makes dendrimers excellent candidates for drug delivery systems (DDS) [8]. Mainly, many recent studies involving DDS using dendrimers have been in the field of neoplastic diseases. Dendrimers are also studied as DDS in other therapeutic fields: anti-inflammatory, antiviral, antibiotic therapies, and in cardiovascular diseases, etc. [9].

Compared to traditional surfactants, when they are used as carriers, dendrimers possess numerous advantages, like a high loading capacity of the drug through numerous functional surface groups and internal cavities, the high bioavailability of the attached drug through covalent or non-covalent bonds, and the high penetrability of biological barriers and cell membranes [10,11,12,13].

Due to their significance in the field of medicine, dendrimers have been studied intensively in the past few years, and because of the extensive number of studies performed regarding this subject, review articles that emphasize several aspects have been published. A great interest has been shown in the biomedical applications of dendrimers, especially for their capacity to be used as targeted drug [7,14,15,16] and gene delivery systems [17,18,19]. Advances in diagnostics that use imaging techniques were made [20,21,22], along with the improvement of treatments for diseases like cancer [23,24,25,26], cardio-vascular diseases [27], inflammatory diseases [26,28,29], and viral and bacterial infections [26,29]. Even though dendrimers possess a wide range of applications in biomedicine, their toxicity was reported as well, for the assessment of limitations in their usage [5,30,31].

In order to obtain a better understanding of these compounds, the synthesis and physicochemical analysis of dendrimers were reviewed as well [31,32]. Different types of dendrimers were compared from biological points of view, thus underlining their properties depending on their composition [33].

The aim of this review is to systematically present the extensive biomedical applications of dendrimers from a pharmaceutical point of view, focusing on the pharmacokinetic and pharmacodynamic advantages they provide. The objectives include: (a) the identification of dendrimer’s applications in therapy and diagnostics, (b) a display of dendrimer types and examples of complexes they form with active substances, grouped by the medical specialty they refer to, and (c) a presentation of dendrimer’s cytotoxicity, the main limiting characteristic of these substances from a medicinal and pharmaceutical perspective.

## 2. Biomedical Dendrimer Profile-Cytotoxicity

In order to introduce a new substance in therapeutics and the diagnostics of human illnesses, its properties have to be well documented. Beside the physicochemical characteristics and the pharmacological profile, the toxicological risk/benefit ratio must also be evaluated. Dendrimers, as biocompatible nanoparticle macromolecules, are used for their unique properties as carriers of other molecular structures, in order to improve the activity and efficiency of an active drug molecule and also to reduce its toxicity.

The special molecular structure of these entities determines the specificity of action. The macromolecules are defined by their molecular weight, highly branched spherical tridimensional structure, and the ability to create a monodisperse media [34].

The selection of the initial central atom, such as carbon, nitrogen and phosphorous, is important in determining the structure of the dendrimer, its branches and its cavities. There are over 100 families of dendrimers [35,36,37].

**Figure 1:**
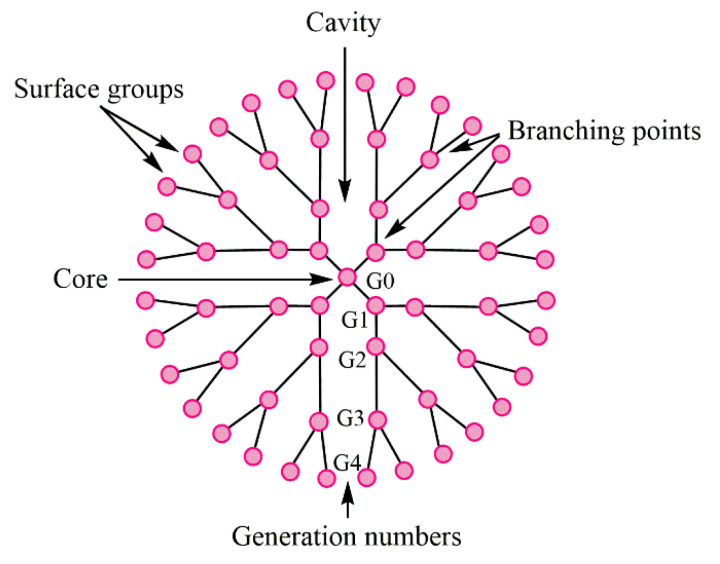
Dendrimer structures—from central structure to periphery, 0–4 generations, linear diametral growth. Adapted from [38], published by Int. J. Nanomed, 2009.

The essential characteristic of these nanomolecules is given by the generation they belong to. There are 13 generations, from G0 to G12, the number representing the branch layers. The schematic structure of these macromolecules is illustrated in Figure 1.

It has been shown that the cytotoxicity of the dendrimer depends on the generation to which it belongs and also on the nature of its surface, given by terminal functional groups. Cytotoxicity was highlighted in cationic, amine dendrimers. Studies also showed a correlation between cytotoxicity and dendrimer generation [39,40]. For example, the cytotoxicity of poly(amidoamine) (PAMAM) and poly(propylene imine) (PPI) dendrimers is directly proportional to concentration and generation, due to the presence of primary amines terminal zones. Grafted polyethylene carbosilane dendrimers are less toxic and so are anionic terminal group dendrimers [41,42,43]. Thus, the surface modification of cationic dendrimers in order to neutralize or completely modify them to anions is directly linked to reduced cytotoxicity [44].

Cationic dendrimers have the ability to interact with negatively charged cell membranes, disrupting their integrity. Punctual defects in the membrane lead to a cascade of events, ending with cell apoptosis [45].

**Figure 2:**
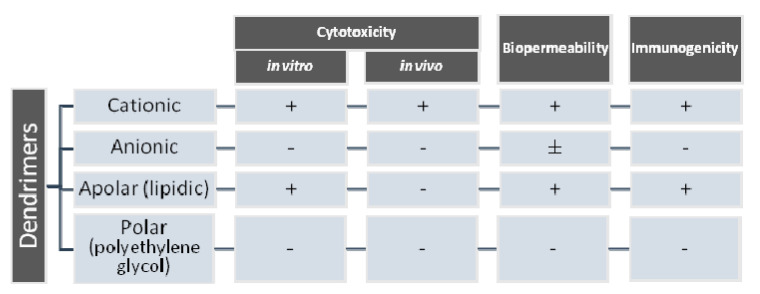
Cytotoxicity, biopermeability, and immunogenicity of dendrimers under the influence of their surface charge; “+” means the presence of an effect, and “−” the lack of an effect [44].

Figure 2 shows the types of dendrimers and how surface charge affects their in vitro and in vivo cytotoxicity, biopermeability and immunogenicity.

The occurrence and modulation of dendrimers’ cytotoxicity was approached by studying various structural modulations, especially in the nanomolecules’ peripheral area, obtaining carbohydrates, acetyl and polyethylene glycol (PEG) derivatives that did not significantly affect cell viability, while maintaining other advantageous features.

**Figure 3:**
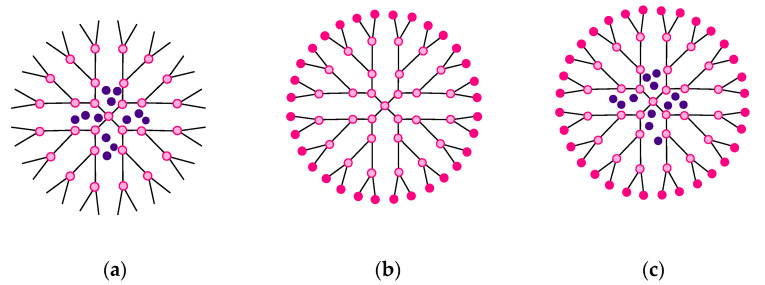
Schematic representation of three ways of complexing or conjugating drug molecules with a dendrimer: (**a**) internal cavities encapsulation, (**b**) peripheral attachment, (**c**) internal cavities encapsulation and peripheral attachment simultaneously. Adapted from [40], published by Biomolecules 2019.

Dendrimers are perfect partners for active pharmaceutical ingredients, due to their structural specificity, which allows the following: (a) inclusion inside the cavities (Figure 3a), (b) attachment of bioactive compounds/drug molecules to the functional groups at the periphery of the dendrimer (Figure 3b), and (c) both of the above—offering encapsulation (internal cavities) and a support for conjugates (on the surface) (Figure 3c). The interaction between drugs and dendrimers is beneficial since it improves solubility, thus improving the absorption and bioavailability of the drug molecule or its cytotoxicity [26,46,47].

Glycodendrimers are a newer type of dendrimers, these modulations leading to a significant decrease in cytotoxicity [2]. The interaction of liposomes and human serum albumin (HSA) with glucose-modified carbosilane dendrimers, from first to third generation (dendrimer 1-3Glu), was evaluated. The interactions with both of the above-mentioned biological structures could not be related to the generation of the dendrimer, but because of the strong interactions with liposomes and the weak ones with HAS, theoretically, cancer cells can be targeted by the overexpression of glucose transporters, thus demonstrating that glucose-modified carbosilane dendrimers can be used as drug delivery carriers in the therapy of cancer [48].

## 3. Biomedical Applications of Dendrimers

Several dendrimers possess intrinsic pharmacodynamic properties [5,6]. In order to be used for their biomedical activity, dendrimers must meet certain conditions, as follows: (a) they must show low toxicity, (b) low immunogenicity, and (c) high permeability, so that they can cross biological barriers, have a proper presence in the systemic circulation and be capable of specific targeting [49]. The limiting characteristic in relation to the medical use of many dendrimers is their cytotoxicity [50].

Dendrimers have been investigated in relation to medical tasks, the targeted release of active molecules, or gene therapy, due to the malleability of their structure which permits the tailoring of their physicochemical properties [51,52,53]. This possibility confers the uniqueness of dendrimers compared to other nanoparticles, their structure on generations (dendrons—branched concentric layers) (offering the possibility of synthesizing dendrimers as monodisperse systems), and the terminal groups offering possibilities for further interaction [54,55,56].

### 3.1. Dendrimers in Drug Therapy

#### 3.1.1. Dendrimers in Antineoplastic Therapy

Cancer is an abnormal proliferation of cells caused by numerous changes under the action of physical, chemical, biological or genetic factors, leading to an imbalance between cell proliferation and apoptosis, and eventually evolving into distant-site invasive cells, causing significant morbidity and mortality. Despite sustained research efforts over recent decades to find effective therapies, cancer continues to be one of the leading causes of mortality [57].

Conventional antineoplastic therapy is associated with many important side effects. Commonly indicated radiotherapy can lead to the development of secondary gene mutations, which could cause complications and future new malignancies. Chemotherapy, immunotherapy and gene therapy are generally characterized by significant nonspecificity, which limits the bioavailability of the drug at the tumor site [58,59].

Chemotherapeutic drugs often have a nonspecific distribution, so that only a small part of the active substance reaches the site of action, and the pharmacokinetic characteristics are directly responsible for the in situ concentration of drug and/or active metabolite [60].

In this context, there is an important concern for the ongoing development of therapeutic strategies that limit as much as possible the side effects of chemotherapy, which is the main method of treatment for neoplastic diseases, because chemotherapy is associated with important toxicity: nephro-, hepato-, hemato- and cardiotoxicity [61].

An increasingly addressed strategy is the use of active molecule carriers, belonging to nanomaterial technology, which aims to improve the target selectivity of drugs in neoplastic cells. Due to their physicochemical properties, nanoparticles pass biobarriers more easily [62].

The carrier capacity of dendrimers offers an advantage and constitutes an important strategy in cancer therapy, dendrimers having the role of useful ligands in transporting the drug molecule to tumor tissue through various biological compartments, while maximizing the pharmacodynamic activity to the targeted site [63]. Drug release from the dendrimer complex is controlled by different mechanisms: degradable spacers at specific sites, and the numeric variation of terminal groups [64]. The structure and specific functionality of dendrimer surfaces, correlated with special properties of these materials, such as high hydrosolubility, allows the encapsulation/conjugation of several entities, either in the central structure or on the surface, which makes dendrimers ideal carriers for various anticancer drugs [65]. Furthermore, the easy way to control the physicochemical properties of dendrimers makes conjugation with them the first option, which ensures optimal distribution and delivery to the pursued action site [66,67].

Dendrimers transport active drug molecules using various strategies: (a) physical interactions based on the inclusion of the active drug molecule in the central structure of the dendrimer through non-covalent associations, hydrogen bonds, hydrophobic or electrostatic interactions [68]; (b) chemical interactions involving the covalent conjugation of drugs with the functional end groups of dendrimers [69], on the other hand, are much more stable. The presence of intramolecular links between dendrimers and molecules commonly used in antineoplastic therapy, such as paclitaxel (PTX), camptothecin (CPT), methotrexate (MTX), 5-fluorouracil (5-FU) and doxorubicin free base (DOX), has been highlighted by numerous studies. These molecules have two major drawbacks: low hydrosolubility and high nonspecific toxicity. Hence, the use of dendrimers is a promising strategy [70,71,72,73].

Numerous examples of drugs conjugated with dendrimers are found in the scientific literature, the main purpose being the increasing of the specificity at the site of action, and at the same time decreasing the systemic toxicity by directing the delivery to the tumor cell.

##### Poly(amidoamine) Dendrimers (PAMAM)

Poly(amidoamine) dendrimers have been frequently conjugated with various drugs indicated in neoplastic diseases:

Doxorubicin (DOX)—used in lung cancer and brain tumors, and has been conjugated to PAMAM dendrimers of the fifth generation (G4). The conjugation was performed via acylhydrazone bonds on the dendrimer surface, with the advantage of increasing the therapeutic efficiency and specificity of action in the lung neoplasm, by directing the pH-controlled DOX-PEG-PAMAM dendrimer [74,75,76,77].

**Figure 4:**
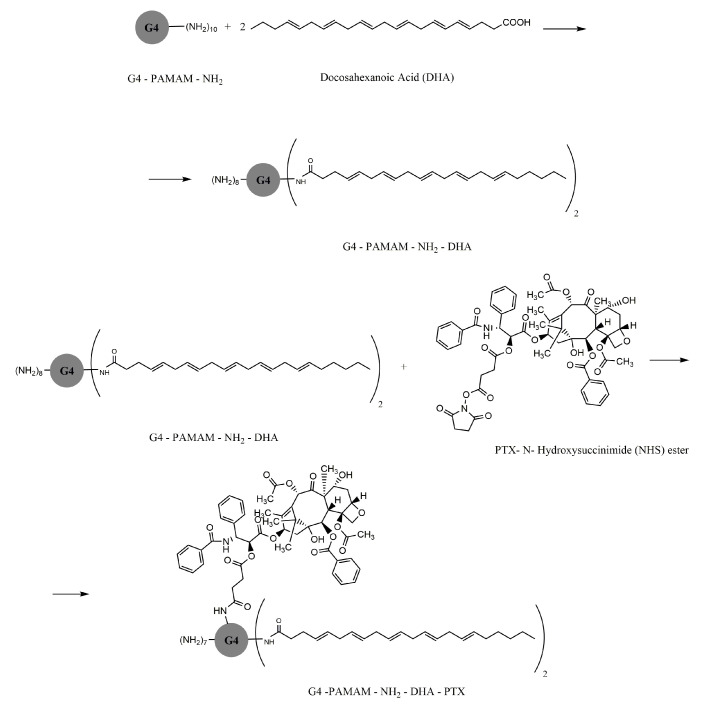
The obtaining of the dendrimeric conjugate G4-PAMAM-NH_2_-DHA-PTX. Adapted from [79], published by Macromol. Biosci. 2017.

Paclitaxel (PTX)—this was conjugated with a PAMAM G4 dendrimer through a glycine–phenylalanine–leucine–glycine peptide linker for the indication of breast cancer. The dendrimeric conjugate increases specificity and cytotoxicity compared to the PTX molecule alone [78]. For the indication of gastrointestinal neoplasm, PTX was conjugated with an omega-3 fatty acid-grafted PAMAM-G4-DHA dendrimer. For this type of conjugation, an increase in pharmacological activity in the upper gastrointestinal neoplasm has been demonstrated, compared to the cytotoxic molecule alone (Figure 4) [79]. The cytotoxic activity in the ovarian neoplasm of a biotinylated PAMAM dendrimer–PTX conjugate was evaluated on OVCAR-3 and HEK293T cells. The complex efficacy was demonstrated compared to the drug alone, and the results obtained from both the experimental evaluation and the statistics confirm that the biotinylated PAMAM NH2 dendrimer—PTX complex improves the pharmacokinetics not only by increasing cell absorption, but also by increasing the release up to 72 h, with reduced cytotoxicity [80]. The association with biotin [81] as a useful ligand in tumor targeting, or with other molecules such as arginine [82], contributes to increasing the specificity of polymers for cancer cells [83]. A biotin conjugate was also perfected for the active cisplatin molecule, obtaining a higher cellular cytotoxicity, with specific targeting to tumor cells in the ovarian neoplasm [84,85].

Docetaxel (DTX)—the surface modification of PAMAM dendrimers is a strategy employed to lower systemic toxicity and to increase tumor targeting [86]. An example of this improvement in efficacy is the case of DTX, indicated in breast cancer: it was conditioned by encapsulating the active molecule of trastuzumab-modified DTX (TZ) on the PAMAM G4 dendrimer surface of the conjugate, using PEG as a linker. This conjugate leads to an increase in the specificity of the DTX-TZ dendrimer systems, compared to the DTX dendrimer on HER-2 positive cells [87,88,89].

Imatinib (IMT)—the PAMAM dendrimer complex of IMT is formed via electrostatic interactions, as in the case of other hydrophobic acid molecules, while also non-polar groups of dendrimer ramifications can act as micelles, increasing solubility [90,91]. Thus, a PEGylated PAMAM G5 dendrimer conjugate of IMT, a drug used in lung cancer, showed increased water solubility [92], and improved targeting and release in neoplastic cells [93].

5-fluorouracil (5-FU)—used in gastric neoplasm as an aptamer conjugate with PAMAM-PEG, it is specifically targeted to MKN45-type neoplastic cells, with increased uptake by neoplastic cells [94,95].

Sunitinib—used in renal neoplasm, is conjugated with an NH_2_-PAMAM-G3 dendrimer through the platinum (II)-based binding system, obtaining the targeting of the active molecule at the neoplastic renal tissue [96].

Cisplatin (CIS)—used in breast and ovarian neoplasm, it was conjugated to the active molecule of herceptin and diglycolamic acid (DGA) in a dendrimeric complex of the type herceptin—DGA-G4-CIS [91]. In vitro studies with herceptin—DGA-G4-CIS in HER-2 human ovarian cancer cell lines have shown that the complexes have outstanding characteristics, decreasing the half maximal inhibitory concentration and increasing neoplastic cell apoptosis, in correlation with the increased cell uptake compared to the dendrimeric conjugate DGA-G4-CIS and the CIS molecule alone. The results confirmed that the association in this conjugate offers a higher specificity of action compared to the two molecules alone [97]. CIS was incorporated into dendrimeric nanoparticles, in which the active molecule is grafted with a polycaprolactone polymer (PCL) via a pH-controlled 2-propion-3-methylmaleic anhydride (PMA) linker; this dendrimeric copolymer PCL-PAMAM-PMA-CIS forms integrative cluster nanoparticles with the addition of PCL-PEG, to prolong action and increase bioavailability [98,99].

In general, higher generation PAMAM dendrimers (4 or higher) have many advantages, such as: (a) high degree of loading of the active drug molecules, through physical or chemical interactions; (b) increased interaction between drug molecules and tertiary amine groups due to the generous space in the dendritic cavities [100]; (c) increased conjugation degree due to the elevated number of terminal groups [101]; (d) high expectations regarding balancing the effectiveness of higher generations of dendrimers and their toxicity [102].

Omega-3 fatty acid [docosahexanoic acid (DHA)]—PAMAM dendrimer: combining dendrimers with different fatty acids is another strategy in developing new conjugated molecules. It has been proven that DHA penetrates cancer cells and manifests tumor inhibitory properties [79,103].

PTX—PAMAM G4.0-NH_2_-DHA-PTX, a DHA-PTX conjugate, is characterized by both improved antineoplastic efficiency and low toxicity [79,101]. This dendrimeric conjugate has demonstrated the potential of the omega-3 fatty acid as a molecule for active tumor targeting and enhancing anticancer activity. The goal of this conjugate was primarily to enhance the bioavailability and cell penetration often associated with PTX [104].

Carboxylated PAMAM dendrimers are generally characterized by covalent bond conjugation, with improved loading efficiency and increased cytocompatibility [105].

**Figure 5:**
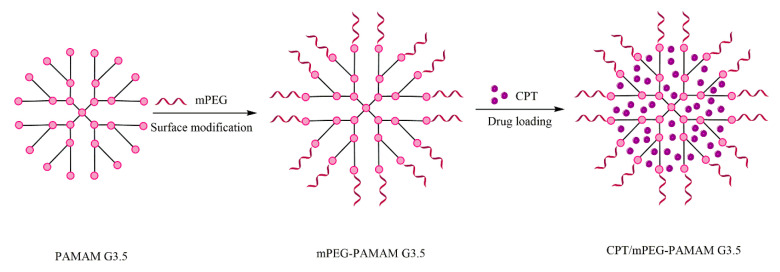
The process of obtaining the carboplatin conjugate (CPT) encapsulated in the mPEG-G3.5 dendrimer. Adapted from [107], published by Int. J. Mol. Sci. 2019.

A CPT—the PAMAM G3.5 dendrimer with carboxylic terminal ramifications, conjugated with poly (ethylene glycol) methyl ether (mPEG), demonstrates the concept of site-selectivity with degradable spacing between the drug and the terminal groups of the dendrimer [106]. Prolonged release of the dendrimer—CPT complex has been demonstrated compared to the free drug, with high cytocompatibility for CPT, so that the active drug is efficiently delivered [107]. In this type of conjugate, the PAMAM dendrimer was selected for the advantages given by the predetermined and controllable structure, by the presence of cavities and compartments for CPT encapsulation and the lack of toxicity induced by the positive load. mPEG conjugation improves the loading capacity of the active molecule and can control the premature release of drug molecules during their transport to the target site, which is demonstrated by the following results [106]. This type of dendrimer conjugate is illustrated in Figure 5.

The combination of an antineoplastic drug with oligonucleotides constitutes a type of dendrimer conjugate that addresses the mechanisms of tumor drug resistance. The tumor invasion and progression of these formations shows an advantage related to increasing therapeutic efficacy through a synergism of the action of the active molecule and the associated oligonucleotide [108,109].

**Figure 6:**
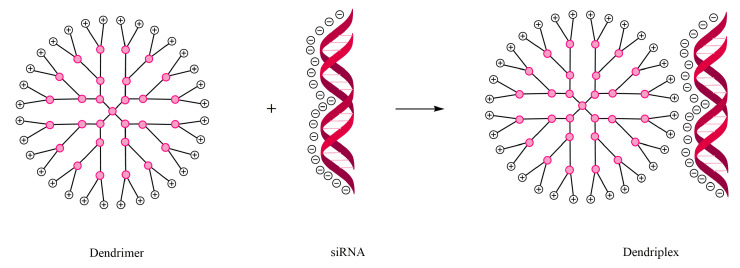
The obtaining of dendriplex (siRNA: small interfering RNA). Adapted from [115], published by Molecules 2020.

There are numerous studies that show promising results for the delivery of nucleic acids to the target site using dendrimers. The dendrimer–nucleic acid complexes are being called “dendriplexes” (Figure 6) [110,111]. The first studies on this type of dendrimer conjugate used PAMAM dendrimers for the delivery of oligonucleotides, and were performed in 1993 by Haensler and Szoka [112]. The formed dendriplexes have a better ability to internalize and release nucleic acids. Examples of such dendrimers include the following: (a) DOX+ siBCL-2, G2, modified with PEG, indicated in B-cell lymphoma [113]; (b) PTX+ siTR3, G2, modified with Plectin-1 peptides, indicated in pancreatic neoplasm [114].

Conjugates were approached to avoid the onset of drug resistance, as in the following examples:

—DOX included in a liposome-encapsulated AS1411 aptamer [Lip (Ap-DOX)]. After the diffusion of the conjugate (Ap-DOX) into MCF-7/Adr neoplastic cells, the Ap-DOX complex binds to the nucleolema and passes to the nucleus. Employing this strategy, DOX·HCl accumulates and leads to the apoptosis of neoplastic cells [116].

—DOX included in a G0-PAMAM dendrimer, which can simultaneously load the MMP-9 shRNA plasmid, leads to obtaining an effective treatment against breast cancer. The co-administration effect of the MMP-9 shRNA plasmid and DOX had a significantly increased efficacy compared to the single drug [117].

##### Poly(propylene imine) Dendrimers (PPI)

Poly(propylene imine) dendrimers are generally characterized by the presence of primary amines terminal groups and tertiary propylene amines inside the PPI structure. The main mechanism by which these dendrimers act is to increase the solubility of the conjugated drug through electrostatic interactions [118]. The main advantages of these dendrimers include: the ease of surface modification, allowing the appearance of high generation dendrimers, as well as the versatility of drug delivery and high functionality [5,119,120].

Melphalan, prescribed in breast cancer, is an example of a dendrimer conjugate obtained by combining the PPI dendrimer with folic acid, which increased the biocompatibility of the active molecule due to the shielding of the cationic folate groups. It increased the inhibition of tumor development and increased survival, particularly G4, G5. The increase in these cationic dendrimers’ generation is associated with higher toxicity [121].

PTX—this was conjugated by modifying the PPI dendrimer with a monoclonal antibody (mAbK1). The monoclonal antibody targets the mesothelin protein, overexpressed in some types of neoplasm. In vitro experiments on ovarian cell line OVCAR-3 demonstrated an effect greater than the PTX or PPI-PTX complex [122].

DOX—a PPI G5 dendrimer conjugate loaded with DOX and dextran, which showed improved absorption on the A549 cancer cell line, as well as a sustained release profile of the active drug molecule, and at the same time decreased hemolytic activity [123].

Methotrexate (MTX)—this has been conjugated through an approach that aims to release the active molecule to the desired site of action, combined with the mechanism of up-regulation and receptor regulation, improving the efficiency of targeting and the transport of methotrexate to cancer cells. Folate-modified PPI G5 dendrimers were synthesized and loaded with MTX and retinoic acid, designed to transport the active drug specifically to tumor cells, characterized by the overexpression of folic acid receptors [124,125].

##### Poly-L-lysine dendrimers (PLL)

**Figure 7:**
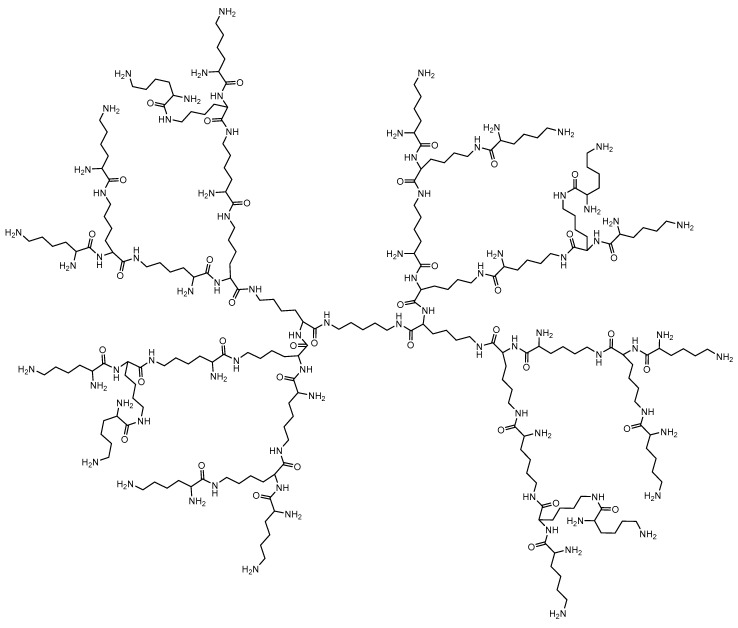
Structure of PLL G3 dendrimer. Adapted from [128], published by Sci. World J. 2013.

Poly-L-lysine (PLL) dendrimers were among the first cationic polymers used in complexes and to release DNA (Figure 7) [126,127]. However, due to their high cellular toxicity and low efficacy (primarily the absence of endosomal escape), PLLs are not widely used for conjugation with various active drug molecules.

DOX—a PLL G6 cationic dendrimer conjugate that was synthesized to improve the penetration of the active DOX molecule into multicellular spheroidal prostate tumor cells, which led to the increased cytotoxic activity of the drug and delayed neoplastic cell development [129].

##### Tryptophan-rich Peptide Dendrimers (TRPD)

**Figure 8:**
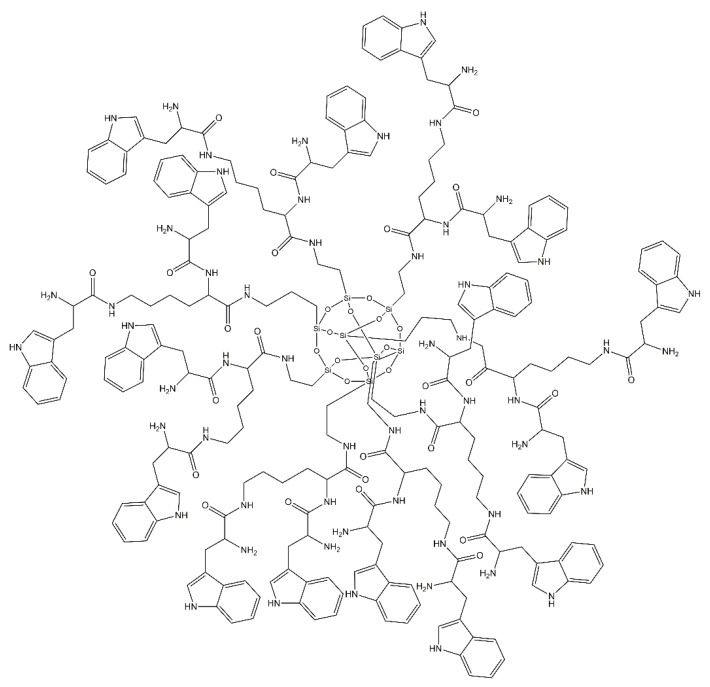
Structural representation of the TRPD dendrimer. Adapted from [130], published by Angew. Chem. 2015.

A new type of tryptophan-rich peptide dendrimer (TRPD) (Figure 8) has been evaluated in antineoplastic therapy. This dendrimer is extremely effective due to its excellent solubility in water, its highly branched structure with several terminal groups, and generally having a spatial structure similar to proteins. TRPD can interact with intracellular DNA, generating efficient supramolecular aggregates. Furthermore, this dendrimer easily penetrates through the tumor cell membrane, exerting extremely high cytotoxic effects on these cells. In general, this type of dendrimer could obstruct tumor cell proliferation in vivo and lead to tumor cell apoptosis [130,131]. The dendrimer approach to cancer therapy is promising with regards to improving the effectiveness of treatment and patient safety. Dendrimers have a special potential, being further evaluated as useful materials in the formulation of cytotoxic drugs and more [132].

##### Phosphorus Dendrimers

**Figure 9:**
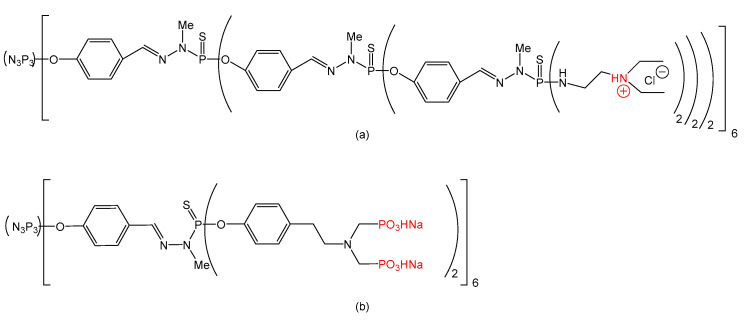
The structures of phosphorhydrazone dendrimers with charged terminal functions: positive (**a**) and negative (**b**). Adapted from [33], published by Molecules 2018.

Phosphorus dendrimers proved to be effective in cancer therapy (direct drug carrier and indirect, inducing the apoptosis of cancerous cells) in both forms: alone and functionalized (on the surface with different drugs or metal complexes of dendrimers). Furthermore, phosphorous dendrimers grafted with fluorophores were synthetized and tested in bioimaging [115]. One of the inorganic branching points of dendrimers is the phosphorous-generating the family of “Phosphorhydrazone dendrimers”. Terminal functions can have positive charges (ammonium) or negative charges (phosphonates) [33]. These structures are presented in Figure 9.

Dendriplexes were synthetized by complexing small interfering RNA (siRNA) with different cationic dendrimers (PAMAM, phosphorous and carbosilane). The tests performed proved that the most effective siRNA carriers are phosphorus dendrimers [133].

**Figure 10:**
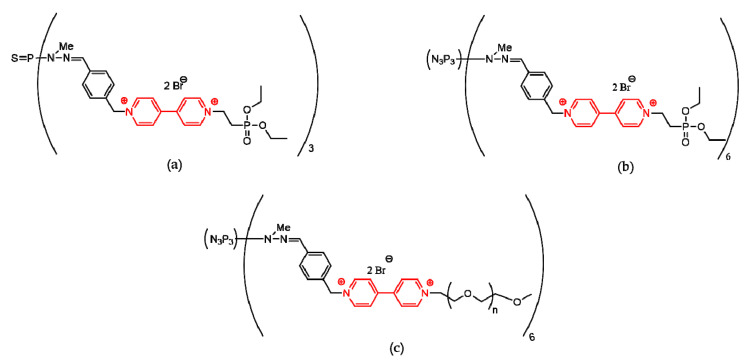
Viologen-phosphorous dendrimers: trifunctional (**a**) and hexafunctionals (**b**) and (**c**). Adapted from [135,136], published by Mol. Pharm. 2012 and Molecules 2013.

Inserting 4,4′-bipyridinium ion in phosphorous dendrimers structures may generate interesting versatile dicationic species (viologen-phosphorus dendrimers or VPDs) that are not limited by insolubility or toxicity [134]. Several VPDs (0 and 1 generations) were synthetized and tested [135,136]. Some of these VPDs presented promising properties during tests (Figure 10).

VPDs (a) and (b) (Figure 10) presented moderate hemolytic activity (under 10%), while (c) presented weak hemolytic activity (around 3%). Tests on B14 Chinese hamster peritoneal fibroblasts revealed a decrease in cell viability in the case of (a) and (b), while (c) was non-toxic. Tests on normal mouse hippocampal cell line (mHippoE-18) proved a low toxicity of (a) and (b). Based on these findings, VPDs are relatively safe, and further investigations into their applications in biomedicine will definitely be performed.

Since more than half of the triple-negative (TNBC) breast cancers are related to epidermal growth factor receptor (EGFR), a therapeutic option is the use of tyrosine kinase inhibitors (TKIs). However, it was proven that ethacrynic acid (EA) (due to its α,β-unsaturated ketone group) has a synergistic effect when combined with TKIs in breast cancer treatment [137]. The synergistic anti-tumor effect of EA and cytokine-induced killer cells (CIK) on hepatocellular carcinoma (HCC) was also demonstrated [138]. EA inhibits the activity of signal transducers and activators of transcription factor 3 (STAT3) on DU145 prostate cancer carcinoma cells. EA binds to the Src homology region 2 (SH2)-containing protein tyrosine phosphatase 2 (SHP2) and protein tyrosine phosphatase 1B (PTP1B), so EA may be used as a treatment/prevention agent of STAT3-dependent tumors [139].

**Figure 11:**
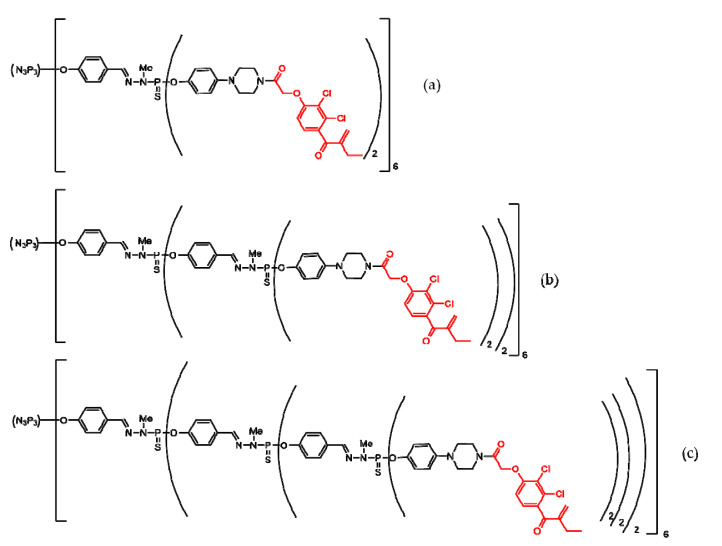
Phosphorhydrazone dendrimers (0 to 3 generations) functionalized with EA: G1 (**a**); G2 (**b**) and G3 (**c**). Adapted from [140], published by Nanoscale 2015.

Several chemical modifications to modulate EA’s antiproliferative activity were performed (both changing several functional groups and by functionalizing phosphorhydrazone dendrimers with EA). The resulting compounds were tested on different cell lines: noncancerous (endothelial progenitor cells—EPC) and concerous (solid tumor, epidermal carcinoma—KB—and liquid tumor, promyelocytic cells—HL60). IC_50_ is half of the maximal inhibitory concentration. All compounds that resulted in the EA functional group’s modification showed to be relative toxic for EPC cells, while three of the phosphorhydrazone dendrimers (Figure 11) proved to be safe for non-cancerous cells (IC_50_ > 100 μM). The anti-proliferative activity of dendrimers on cancerous cell lines was promising, and was demonstrated to increase together with dendrimer generation (G1 to G3), probably due to the number of EA moieties on the surface of dendrimers (12 for G1, 24 for G2 and 48 for G3). The percentage inhibition of dendrimers at 10 μM ranged between 66% and 75% on the HL60 cell line, and between 91% and 93% on the KB cell line. Further, the IC_50_ of the G3 dendrimer was 110 times lower [140,141].

**Figure 12:**
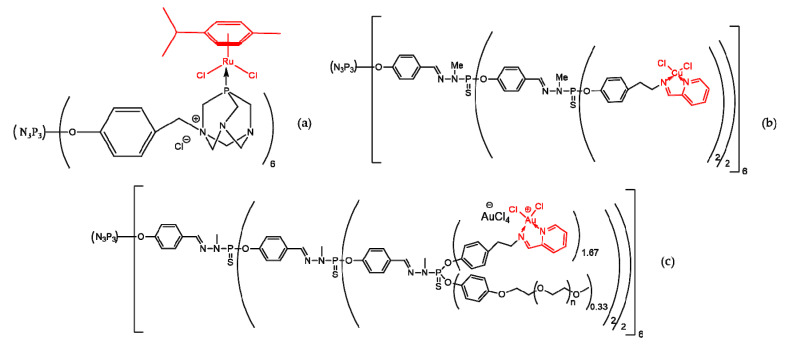
Phosphorous dendrimers functionalized with a rutenium complex of phosphatriazaadamantane (**a**), and with a pyridine imine ligand complexed with Cu (II) (**b**) and Au (III) (**c**). Adapted from [142,143,144], published by Inorg. Chim. Acta 2018 and Mol. Pharm. 2013 and 2017.

Metallic derivatives (organometalics and metal complexes) are used in cancer therapy. Three examples of this type of compound, based on phosphorous dendrimers, are 0 generation functionalized with a rutenium complex of phosphatriazaadamantane, third generation phosphorhydrazone functionalized with a pyridine imine ligand and complexed with Cu (II), and third generation PEG-ilated phosphorhydrazone functionalized with a pyridine imine ligand complexed with Au (III) (Figure 12).

The efficiency of the ruthenium dendrimer in interacting with DNA and generating a relaxed form (relaxed DNA) was proven by the determination of Ri value (concentration which leads to the complete conversion of supercoiled DNA to the relaxed form of DNA). The Ri value of the ruthenium dendrimer was at least five times smaller in comparison with cisplatin [142].

Cu (II) complexed dendrimer showed an anti-proliferative activity of at least 80%, at a concentration of 1 µM, on KB and HL60 cell lines, and an IC_50_ a few times higher than that of the uncomplexed dendrimer (on several non-cancerous cell lines), proving its selectivity [143].

The Au (III) complexed dendrimer proved to be effective on several cancerous cell lines: KB—IC_50_ = 5.5 µM; HL60—IC_50_ = 1.7 µM; MCF7 (human breast adenocarcinoma cell line)—IC_50_ = 2.5 µM and PC3 (prostatic small cell carcinoma). The Au (III) complexed dendrimer also seems to be safe for non-cancerous cells (EPC—IC_50_ > 1000 µM) [144].

There are several biomedical applications of Rose Bengal (RB), especially in cancer treatment and antimicrobial therapy (photo-activation and sono-activation). Photodynamic therapy is another application of RB. RB is a type II photosensitizer that, once activated, generates singlet oxygen, but has the main disadvantage of a superficial penetration into tissues due to its tendency to aggregate in aqueous solutions [145].

**Figure 13:**
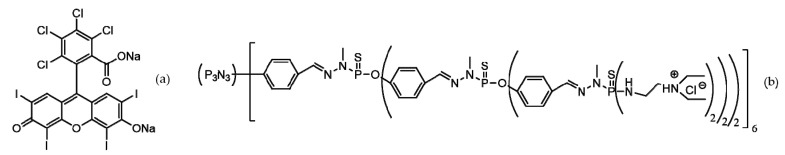
Structures of: RB (**a**) and cationic phosphorous third generation dendrimer (**b**). Adapted from [146], published by Mol. Pharm. 2017.

A complex between RB and a cationic phosphorous third generation dendrimer (Figure 13) was synthetized and tested versus RB alone, in terms of singlet oxygen production, cellular uptake and phototoxicity, on three basal carcinoma cell lines. The complex was able to generate more singlet oxygen than RB alone, the cellular uptake was higher for the complex versus RB alone, and the cell viability was over 90% with no irradiation on all cell lines. The after-irradiation viability of all cell lines was significantly low when comparing the complex with RB alone (at 0.5 µM, RB generated a cell viability of 90%, and the complex generated a cell viability of only 7%) [146].

#### 3.1.2. Dendrimers in Anti-Inflammatory Therapy

Nanotechnology is a technological approach with a wide range of potential applications, with a significant impact on medical practice. Thus, nanomedicine, as one of the most important fields of application of nanotechnology, combines nanotechnology with medical therapeutics, and includes highly specific drugs for clinical practice [147].

The interest in the studying of dendrimers as carriers of active non-steroidal anti-inflammatory drugs (NSAIDs) is increasing. NSAIDs are one of the most widely used classes of drugs, but their use is often limited because of the considerable level of toxicity and associated side effects. Most NSAIDs are hydrophobic molecules, poorly soluble, and have low bioavailability [148]. To improve the solubility of this class of drugs, numerous studies have been performed using water-soluble dendrimers, such as PAMAM or PPI dendrimers [149,150,151,152]. Due to the presence of amino-terminal groups in these dendrimers, the solubilization of hydrophobic NSAID molecules is possible by using encapsulation technologies, while improving the bioavailability of NSAIDs as well [67,153]. The main mechanism of interaction between the active NSAID molecule and the dendrimer takes place between the dendrimer’s amino groups and the NSAIDs carboxyl groups [154,155].

In addition, the use of dendrimeric nanostructures in inflammatory diseases is advantageous due to the intrinsic anti-inflammatory activity of these molecules. They can be prescribed for the treatment of diseases such as rheumatoid arthritis, atherosclerosis and other associated diseases [26,156,157].

Ibuprofen is a NSAID frequently indicated in various diseases for its analgesic and anti-inflammatory properties. The molecule’s hydrophobia limits its bioavailability after oral administration, especially in high doses [158]. The use of this NSAID is restricted because of the side effects in the gastrointestinal tract (ulceration, bleeding and perforation) [159,160]. Structurally, it is a derivative that contains a terminal acidic group that accentuates its effectiveness, but is also responsible for some side effects, especially gastric ulcers [161,162]. Conjugation with a PAMAM G4 dendrimer significantly improved its solubility in direct proportion to the concentration of the dendrimer, and in inverse proportion to the temperature [163].

**Figure 14:**
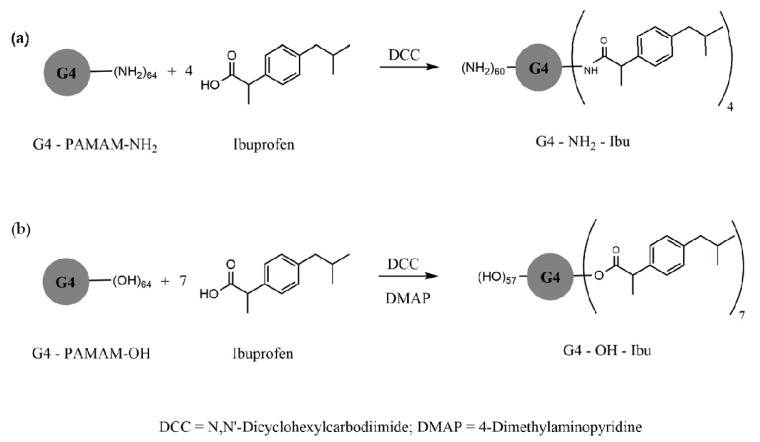
The obtaining of PAMAM G4 dendrimeric conjugates with the active molecule of ibuprofen: (**a**) Ibuprofen–G4-NH_2_ conjugated through terminal amino groups; (**b**) Ibuprofen–G4-OH, conjugated via ester bonds. Adapted from [165], published by Brazilian J. Pharm. Sci. 2013.

**Figure 15:**
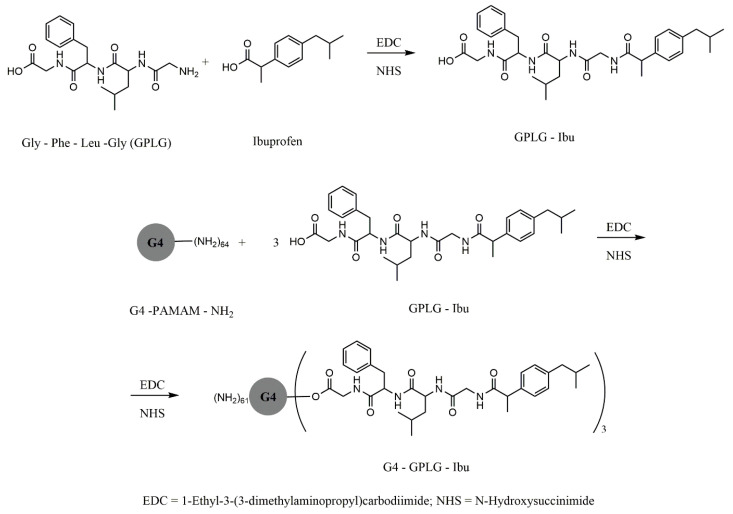
Dendrimeric conjugate Ibuprofen–Gly-Phe-Leu-Gly G4. Adapted from [162], published by Pharm. J. 2011.

Figure 14 and Figure 15 exemplify several types of dendrimer—ibuprofen G4-PAMAM conjugates with ester, amide and peptide linkers. Conjugates via amine end groups have good hydrolysis stability, and ester conjugates release the active pH-dependent ibuprofen molecule from 3% (pH = 5) to 38% (pH = 8.5) [164,165].

Other types of dendrimeric conjugates were synthesized with ibuprofen, via the amino acid linker glycine-phenylalanine-leucine-glycine, such as the PAMAM-NH_2_ G4 amine conjugate.

PAMAM dendrimers increase the solubility of the drug more than sodium dodecyl sulfate micelles, when ibuprofen is in the ionized state, both by encapsulation in the inner cavities through hydrophobic interactions and by surface attachment through electrostatic interactions [165,166].

**Figure 16:**
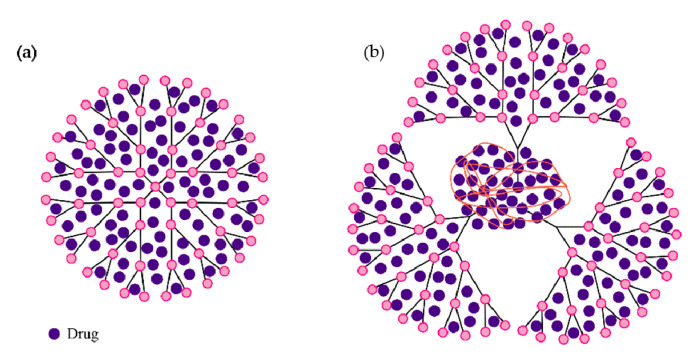
Possible interactions between NSAIDs and (**a**) PAMAM- and (**b**) PPO-PAMAM dendrimers. Adapted from [167], published by Int. J. Pharm. 2013.

To increase the sustained loading/release capacity of drugs, Koc and Senel synthesized and evaluated a different type of PAMAM dendrimer with increased efficiency for NSAID delivery to the site of action, by introducing a propylene oxide residue (PPO) into the central structure of the PAMAM dendrimer. These dendrimers were conjugated with molecules of ketoprofen, ibuprofen and diflunisal, and the effects of the concentration of the active substance, as correlated with the size, the central structure, and the generation of the dendrimer on the water solubility of these NSAIDs, were evaluated. The solubility of these drugs increased along with the generation of the dendrimer, because of the increased size of the nucleus and because the internal structure of the dendrimers corresponds to an optimal interaction with the drugs, in the PP-PAMAM dendrimer, compared to the simple PAMAM dendrimer (Figure 16) [167].

The use of PAMAM dendrimers conjugated with NSAIDs for transdermal administration was also evaluated. The solubility was improved, thus increasing transdermal penetration. This approach has been studied for several NSAID molecules, like indomethacin, piroxicam, ketoprofen and diflunisal, showing an increase in the bioavailability of these drugs [168].

Conjugates between ketoprofen and diflunisal with PAMAM G5 dendrimers were synthesized and evaluated. These complexes have shown three times higher permeability compared to ketoprofen and diflunisal formulations alone [147].

#### 3.1.3. Dendrimers in Antibacterial Therapy

Antimicrobial therapies use chemotherapeutic agents that exert their action on microorganisms by using several mechanisms, like the inhibition of the synthesis of the cell wall, proteins, nucleic acids or other metabolic pathways, or by interfering with the integrity of the membrane [169]. They are useful in the control of bacterial infections, but they have some limitations, such as that the antimicrobial activity spectrum can be restricted, there are issues of the safety and tolerability of the drug [170], and improper administration can lead to unwanted reactions (such as side effects, allergies or toxicity [171]), the inefficient distribution and delivery of drugs, and bacterial resistance to antibiotics [172,173].

Currently, over 70% of the microorganisms that cause infections are resistant to at least one of the most commonly used antibacterial drugs. The emergence of vancomycin-resistant enterococci, which are resistant to many commonly used antibiotics, and methicillin-resistant *Staphylococcus aureus* has led to an increased interest in studying the possibilities of overcoming this limitation. Over 40% of nosocomial *S. aureus* strains are resistant to methicillin, and less of them are resistant to vancomycin [174,175,176].

One way to overcome the challenges posed by antibiotics is using nanotechnology. Many types of nanoparticles, such as polymeric micelles, biodegradable polymeric nanoparticles, fullerenes, nanocapsules, nanogels, nanoliposomes, solid lipid nanoparticles, metal nanoparticles and dendrimers, have been used as drug delivery systems [177].

The encapsulation of antibiotics in dendrimeric systems can improve their therapeutic efficacy and reduce their side effects to a minimum. The main objectives in the design of dendrimers as delivery systems are the control of particle size, the properties of the surface, the functionality and branch length/density, and the release of drugs in order to obtain the wanted effect at the marked site of action [178]. The active molecules can be condensed inside the dendrimers, physically adsorbed, or chemically attached to the surface of the dendrimer. These structures lead to an improvement in the pharmacokinetic and pharmacodynamic properties of drugs, and can be used in combination with traditional drugs [179].

One of the most studied dendrimers for the release of antibacterial drugs is the PAMAM dendrimer, because of its hydrophilic properties which are derived from the large number of surface functional groups, thus the conjugation with antibacterial drugs is performed easily. When these dendrimers interact with water-soluble antibiotics, an improvement in the antibacterial properties can be observed. The biocompatibility of substances can be improved by the replacing of the PAMAM dendrimers amino-terminal groups with PEG or lauroyl chains.

The fluoroquinolones (nadifloxacin and prulifloxacin), conjugated with PAMAM G4 dendrimers with ethylene-diamine surface groups (64 NH_2_ groups), demonstrated a significant increase in their antimicrobial activity and water solubility [180,181].

**Figure 17:**
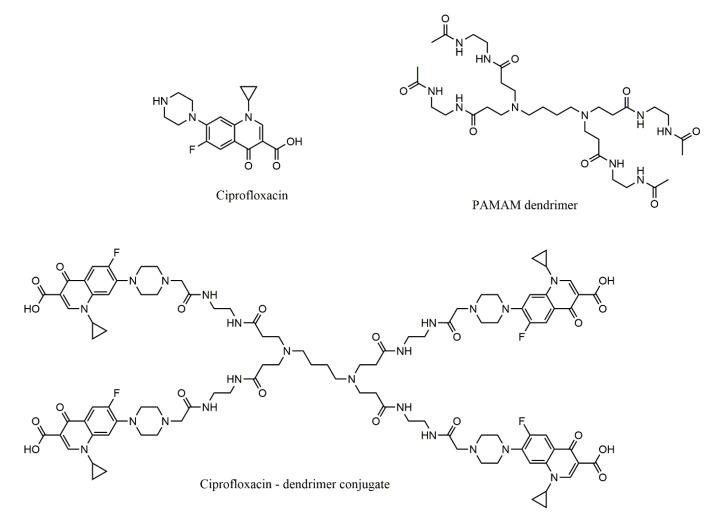
Structures of the active molecule ciprofloxacin, PAMAM dendrimer and ciprofloxacin-dendrimer conjugate. Adapted from [182], published by Molecules 2020.

Ciprofloxacin was loaded on simple PPI and PEGylated PPI dendritic structures in order to assess the resistance of *Staphylococcus aureus* and *Cryptococcus pneumoniae* strains to it. The dendrimer loaded with ciprofloxacin had a significantly higher antibacterial activity than each of the components alone, which demonstrates a synergy of action between ciprofloxacin and the dendrimer [182]. Figure 17 shows the structure of the PAMAM dendrimer conjugate with four molecules of ciprofloxacin.

Anionic dendrimeric polymers conjugated with different molecules with antibacterial activity have been less studied. A 2019 study focused on dendrimeric conjugates with levofloxacin that exerts a known activity on Gram-negative bacteria, such as *Escherichia coli* and *Proteus hauseri*, and Gram-positive bacteria, such as *Staphylococcus aureus* [183]. The synergistic effect of the maltose PPI glycodendrimer (PPI-G3-DS-Mal) with levofloxacin was highlighted. To each amino-terminal surface group of the dendrimer, two maltose units were attached. The PPI-G3-DS-Mal glycodendrimer and a G4 phosphorus anionic dendrimer, with 96 carboxyl surface groups, enhanced the antibacterial properties of levofloxacin, thus a lower dose of antibiotic was administered. This study was the first one to use anionic dendrimers of phosphorus alone or conjugated with levofloxacin as antibacterial agents.

Intense global concern has been identified regarding the prevalence of the antibiotic resistance of some bacterial strains, in particular microorganisms under the acronym ESKAPE: *Enterococcus faecium*, *Staphylococcus aureus*, *Klebsiella pneumoniae*, *Acinetobacter baumannii*, *Pseudomonas aeruginosa* and *Enterobacter spp.* [184,185]. These microorganisms have developed mechanisms of resistance to common antibacterial treatments, and are considered to be critical and high priority by the World Health Organization [186,187].

In the context of the development of antibiotic-resistant bacterial strains, possible dendrimeric conjugates with different antibiotics were evaluated to improve the antibacterial activity of these drugs [188,189,190,191]. The dendrimers themselves possess their own antimicrobial activity, demonstrated by numerous studies [192,193]. The most used dendrimeric polymers in various therapeutic applications are the PAMAM and PPI dendrimers [194]. Due to the multifunctional groups in the structure of dendrimers, they can be conjugated with antibiotics, thus enhancing the activity of both compounds. The antibiotics can be controlled and released from the dendrimer under the action of various factors, like light, pH or temperature. These dendrimeric conjugates are usually from generations two or three, and are known as PAMAM-NH_2_ dendrimers. Moreover, carbosilane dendrimers from higher generations (G4–G6) have higher toxicity, which generally limits their use as conjugates with antibiotics [192,195,196,197].

An example of how the size of the dendrimer can affect antibacterial properties is based on a G2 nanodendrimeric conjugate with erythromycin, whose antibacterial activity was evaluated, and compared to the activity of the free antibiotic against bacterial species of *Pseudomonas aeruginosa*, *Staphylococcus aureus*, *S. saprophyticus* and *S. epidermidis*. The antibacterial activity of the dendrimeric conjugate merged with the nanodendrimers potential of targeting, providing a sustained delivery of the drug within the cell. The erythromycin-conjugated nanodendrimer showed significantly higher antibacterial activity compared to the free erythromycin against Gram-positive and Gram-negative bacteria. Minimum bactericidal concentration (MBC), in terms of the μg/mL of the dendrimer in comparison to that of the erythromycin alone, was four times lower on *P. aeruginosa* and *S. saprophyticus*, two times lower on *S. aureus* and 16 times lower on *S. epidermis*. Since the drug loading percentage was 35.2%, the ratios of the MBC of erythromycin alone to that of the dendrimer, in terms of μM, are even larger [198]. The G2 and G3 PAMAM dendrimers with amino or hydroxyl surface groups conjugated with tobramycin were developed in the same manner. The PAMAM dendrimers with amino surface groups had the most intense antibacterial activity. These groups are protonated, thus promoting the disruption of anionic bacterial cell membranes by electrostatic interactions, which are necessary for the antibacterial mechanism [199,200].

An increase in the solubility of a developed erythromycin conjugate with G2 and G3 PAMAM dendrimers, with amino surface groups, used as a topical hydrogel, was observed [200].

**Figure 18:**
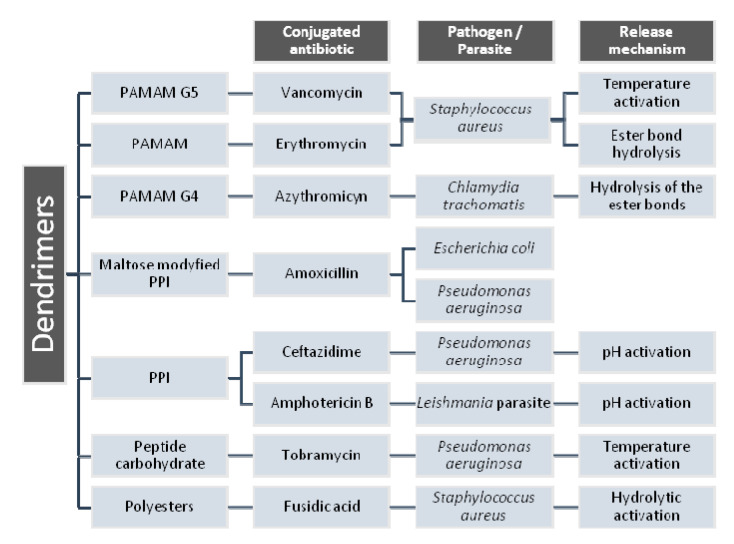
Dendrimers conjugated with antibiotics [186,201].

Other examples of dendrimer conjugated with antibiotics are presented in Figure 18 [186,201].

The emergence of bacterial species able to synthetize extended spectrum beta-lactamase (ESBL) and Gram-negative bacteria-producing carbapenemases is considered a challenge for clinical practice. The development of new strategies for the treatment of infections caused by drug-resistant pathogens is considered a global emergency. Thus, there is an intense concern for the development of alternative treatments, like the combination of antibiotics with antibacterial peptides, bacteriophage species and nanoparticles [184]. Combinations of two or more therapies are used to overcome individual limitations, offering an alternative solution, or extending the lifespan of current antimicrobial agents.

#### 3.1.4. Dendrimers in Antiviral Therapy

Human health can be affected by various agents, like bacteria, viruses, fungi and various parasites. Of these, viruses can reproduce inside living cells by using their enzymatic systems [202,203].

**Figure 19:**
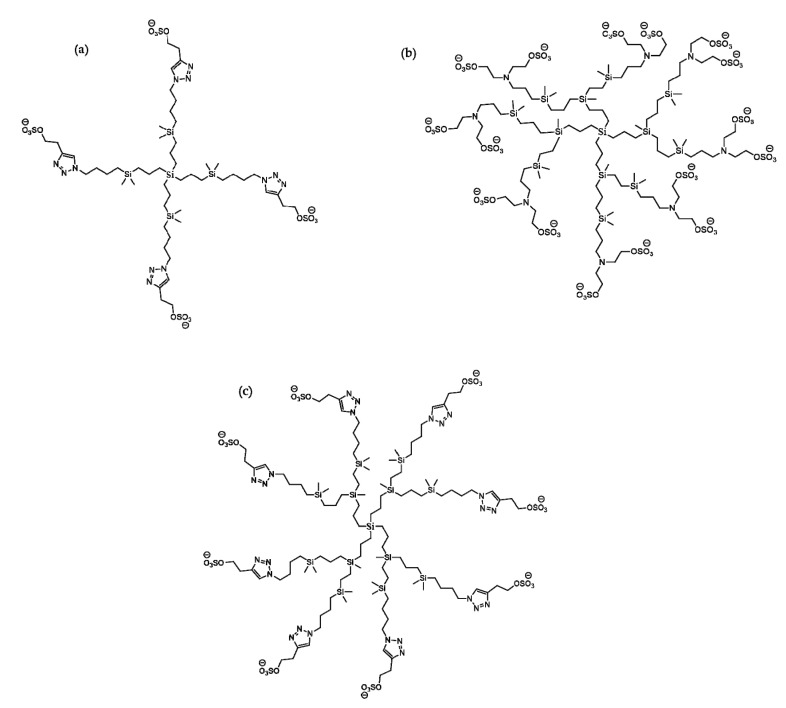
The structures of PCDs used for conjugation with different anti-HIV agents: (**a**) G1-S4, (**b**) G2-S16, (**c**) G3-S16. Adapted from [206], published by J. Nanobiotechnol. 2019.

In antiviral therapy, numerous studies have been performed for the development of dendrimeric conjugates with active substances, which offer multiple advantages, such as increased specificity and bioavailability, prolonged half-life, and the reduced toxicity of the drug [204]. In the last decade, in anti-HIV therapy, nanotechnology using polyanionic carbosilane dendrimers (PCD) has been a promising approach in improving the characteristics of antiretroviral drugs, using dendrimeric nanoparticles with dimensions between 1 and 40 nm [205] and different generations G1-S4, G2-S16 and G3-S16 [206]. These compounds are characterized by the sulfonate groups in the peripheral structures, as follows: G1-S4 PCDs have four peripheral sulfonate groups, and G2-S16 and G3-S16 have 16 groups [207,208]. The number of repeated layers of atoms of silicon determines the generation of dendrimers. These structures are presented in Figure 19.

Tenofovir (TFV) and maraviroc (MRV) were evaluated in comparison with their dendrimeric conjugates. The results showed that the conjugates can provide a higher efficacy, thus lower doses can be used in order to obtain the same clinical effects, which can minimize the toxicity and the emergence of drug-resistant mutations of the virus. At the same time, the synergism and the increase of antiviral potency were demonstrated. Several dendrimers (of first (G1) and second (G2) generations) alone and in combination were tested: G1-S16 (silicon core, 16 sulfonate surface groups), G1-NS16 (silicone core, 16 naphthyl-sulfonate surface groups), G2-S16 (silicon core, 16 sulfonate surface groups), G2-STE16 (silicon core, 16 sulfonate surface groups) and G2-S24P (polyphenoxo core, 24 sulfonate surface groups). Several triple combinations of dendrimer, TFV and MRV were also tested on specific cell lines. The results were compared in terms of EC_50_ (half maximal effective concentration) and combination index (CI). CI > 1.1 indicates antagonism and 1.1 > CI > 0.9 means additive activity, while CI < 0.9 is interpreted as a synergistic effect. The following synergism levels were considered: 0.9 > CI > 0.85 (slight), 0.85 > CI > 0.7 (moderate), 0.7 > CI > 0.3 (synergism), 0.3 > CI > 0.1 (strong), and CI < 0.1 (very strong). G2-STE16/TFV/MRV (molar ratio 10:5:1) showed an EC_50_ 6000 times smaller for MRV and the synergistic interactions of components (CI range of 0.03-0.46). G2-S24P/TFV/MRV (molar ratio 10:5:1) has an EC_50_ 1930 times smaller for MRV, and exhibits strong synergism inhibition (CI range of 0.08-0.57). G2-S16/TFV/MRV (molar ratio 10:5:1) is able to reduce 3000-fold the EC_50_ for MRV, and this combination has strong synergy (CI range of 0.11–0.44). Thus, the best combination is the third, due to its best potentiating effect and synergistic effects [209,210].

**Figure 20:**
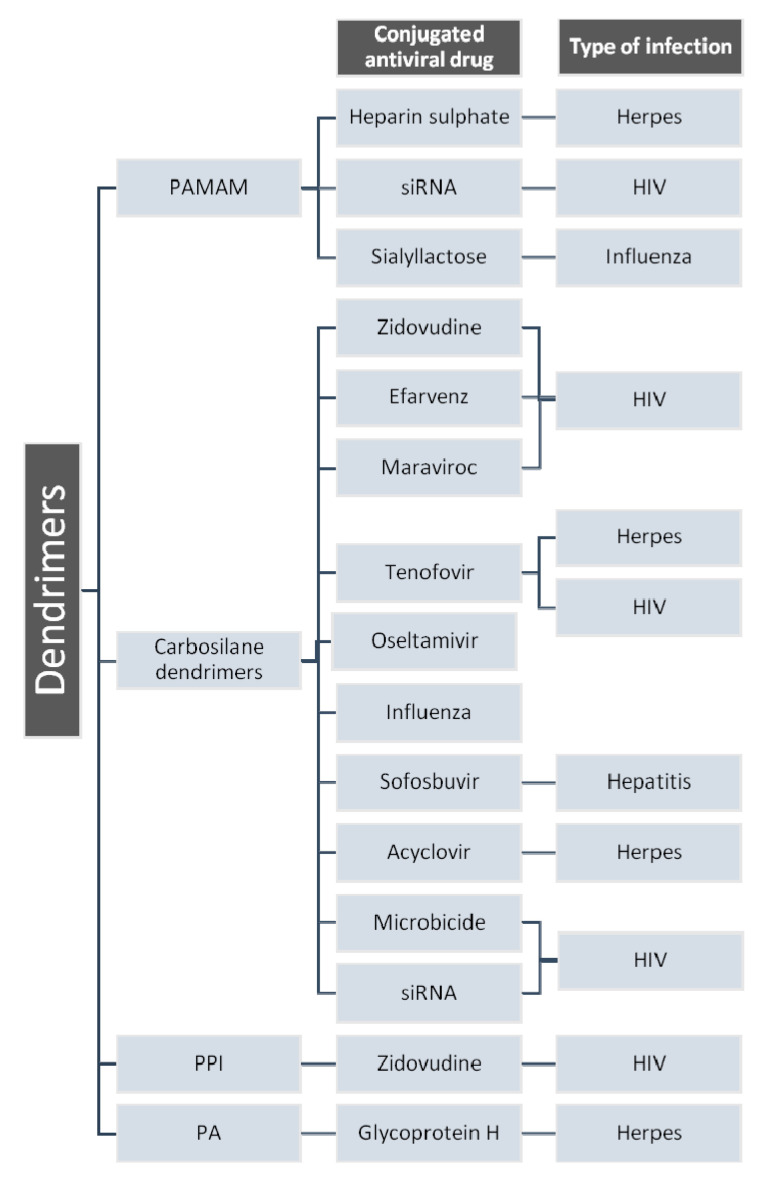
Dendrimers used in antiviral therapy [204].

Some important dendrimers are used in antiviral therapy. Some of them are presented in Figure 20.

**Figure 21:**
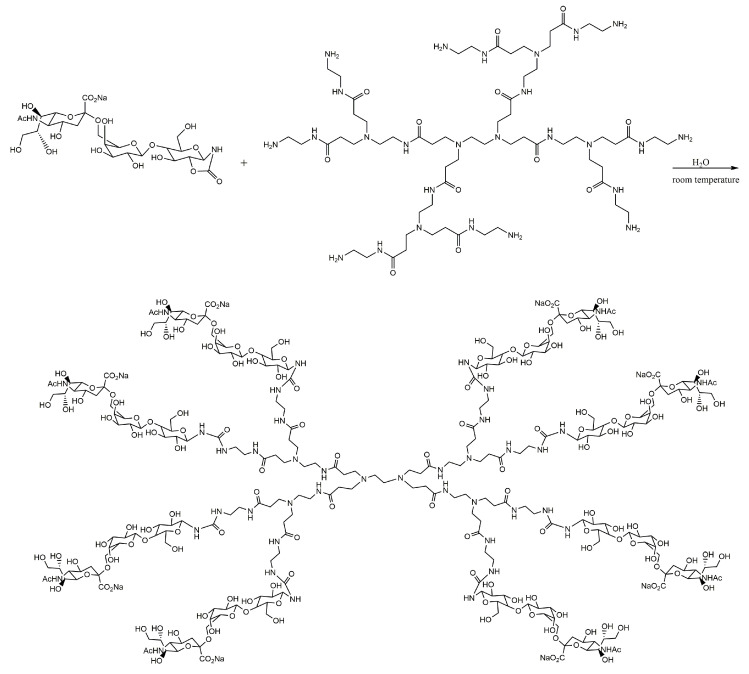
Synthesis of the (6SL) 8-PAMAM dendrimer. Adapted from [211], published by Sci. Rep. 2020.

The inhibition potential of 3′-sialyllactose (3SL), or 6′-sialyllactose (6SL) molecules conjugated with PAMAM dendrimers, was studied for human and avian influenza virus strains. The mechanism of inhibition of viral hemagglutination was demonstrated in a comparative study. Thus, human viral strains can be inhibited by conjugates of the type 6SL, and less by conjugates of the type 3SL. In the avian viral strains, inhibition occurs to an increased extent under the action of dendrimeric conjugates 3SL [211,212]. The trisaccharides 3SL and 6SL were derivatized by a cyclic carbamate reduction mechanism, which allowed the conjugation of sialyllactoses with primary amines in PAMAM dendrimers (Figure 21). (3SL)4-, (3SL)8- and (6SL)4-PAMAM dendrimers were synthetized in the same manner from PAMAMs with an ethylenediamine core (both tetravalent and octavalent).

#### 3.1.5. Dendrimers in Cardiovascular Therapy

In cardiovascular pathologies, due to the low bioavailability of drugs, dendrimeric conjugates have been studied.

The renin–angiotensin–aldosterone system (RAAS) is involved in cardiovascular pathologies. Its excessive stimulation can cause vascular and cardiac hypotrophy and fibrosis. Cardiac remodeling, ventricular disfunctions and heart failure can be caused by the overexpression of angiotensin II, which is a peptide involved in RAAS. The inhibition of its activation can be involved in the prevention and treatment of cardiovascular diseases. Most of the adverse effects of angiotensin II are mediated by the angiotensin II receptor, type 1 (AT1R). PAMAM dendrimers were used as carriers for siRNA to reduce the expression of AT1R in a rat ischemia-reperfusion (IR) model [27].

An siRNA delivery system was studied. It was comprised of two cell-penetrating peptides, oligo-arginine and a transactivator of transcription, linked to a G4 PAMAM dendrimer through a PEG crosslinker [213]. The loading of siRNA in this delivery system had effective downregulation effects on the expression of AT1R in cardiomyocytes in vitro. In vivo, the delivery of siRNA prevented the increase in the AT1R levels, and it improved the recovery of the cardiac function after IR injury, compared to the groups treated with saline solution or dendrimers alone [27,213].

Due to its low water solubility over a pH range of 4–13, nifedipine possesses a low bioavailability in the human body. PAMAM dendrimers from G0 to G3, with amine or ester surface functional groups, increased the water solubility of nifedipine at a pH of 7. The ester surface functional groups had a greater efficiency than the amine ones. Thus, PAMAM dendrimers could act as solubilizers for nifedipine, in order to increase its therapeutic effects [27].

**Figure 22:**
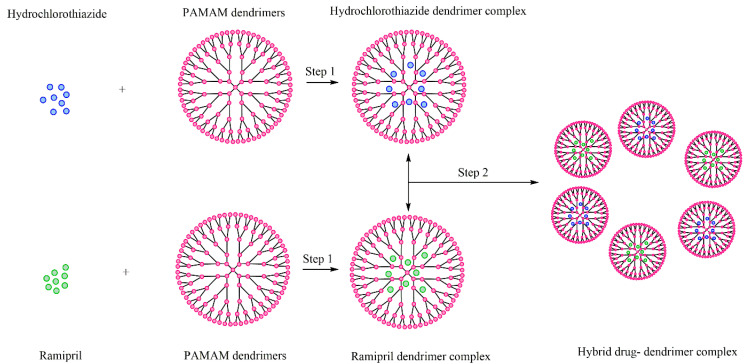
Obtaining of a hybrid drug-dendrimer complex in 2 steps. Adapted from [214], published by Eur. J. Pharm. Sci. 2017.

The combination of ramipril and hydrochlorothiazide has proven to be effective in the treatment of hypertension. Using PAMAM dendrimers, the combination of these two drugs was performed in two steps. The first step provided the entrapment of both drugs separately, and the second one provided the mixing of these complexes in a single formulation, thus obtaining a hybrid drug–dendrimer complex (Figure 22) [214].

The obtained hybrid Ramipril-hydrochlorothiazide dendrimer complex formulation can provide a higher drug loading, and a better solubility, dissolution and stability, to the drugs, thus improving their clinical applications [214].

The low solubility and bioavailability of candesartan cilexetil suggested this drug for dendrimer entrapment studies. The solubility of the drug could be enhanced by increasing the concentration and the generation of the used PAMAM dendrimer. Regarding the involvement of the surface functional groups in the solubility of the complex, the dendrimers that possessed carboxyl or tris(hydroxymethyl)aminomethane (TRIS) groups had higher solubility than the ones with amine surface groups [215].

In vitro studies of PAMAM dendrimers showed an increase in the solubility and dissolution of simvastatin. This depended on the pH of the solution, the concentration of the dendrimer and its available functional groups. Simvastatin-dendrimer complexes can provide the controlled release of the drug as well. The PEGylated, PAMAM-G4 dendrimers showed increased solubility, dissolution, stability and biocompatibility, and slower release of simvastatin, in comparison with the non-PEGylated dendrimers [216].

### 3.2. Dendrimers in Imaging Diagnostics

Nanomedicine and the use of feasible materials in this branch has an increasingly important role in numerous clinical applications, in the supply of drugs and in molecular imaging, through the use of biomarkers and biosensors. A priority in several research areas in which nanotechnology is extremely important is the drug therapy used for the obtaining of specific targets and methods for early diagnosis.

Nanomaterials (NM) are structures with dimensions of no more than 100 nm, used as excipients that can be important in the solving of the bioavailability of active substances issues [217,218,219,220].

Nanotechnology-based imaging is a promising field of interest for overcoming some limitations to the use of imaging agents, and especially for enhancing permeation and retention (EPR), because of the possibility of improving the specificity and the sensitivity of imaging [220].

The advantage of using NM imaging agents is that they can penetrate and accumulate specifically in tumor tissue through the EPR effect, due to dysfunctional vascularization and lymphatic drainage in the tumor microenvironment [101,221]. The EPR effect, also called “the passive tumor targeting effect”, can augment the concentration of the imaging agent in the tumor, thus increasing the sensitivity and the resolution of the image [222,223].

Systems obtained via the self-assembly of supramolecular nanostructures formed by amphiphilic dendrimers represent innovative and efficient drug delivery systems [224]. The use of these amphiphilic dendrimers offers the advantage of well-defined structures and the stability of dendrimers in generating nanostructures of appropriate dimensions, and the possibility of high drug loading [225,226].

**Figure 23:**
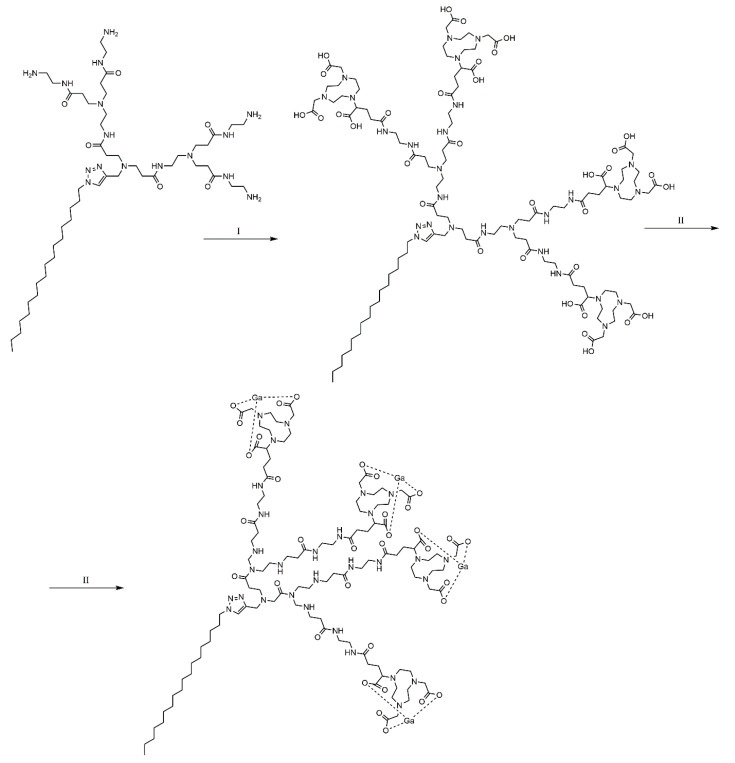
Self-assembly of the 1,4,7-triazacyclononane-1,4,7-triacetic acid (I) amphiphilic dendrimer with the radioisotope [^68^Ga] Ga^3+^ at the terminal branched groups (II). Adapted from [220], published by Proc. Natl. Acad. Sci. USA 2018.

A conclusive example is gallium-68 [^68^Ga] as a high positron emission radioisotope, frequently used in positron emission tomography (PET). This radioisotope has a half-life of 68 min, enough time to obtain images, but the disadvantage is that it does not provide radiation protection for both patients and medical staff [227]. To avoid these disadvantages, gallium was chelated with 1,4,7-triazacyclononane-1,4,7-triacetic acid, which has increased stability in vivo. This dendrimer is able to assemble itself into a complex of nanomicelles that are uniform and stable. It can accumulate with high efficiency in tumors by means of the EPR effect, and provide images of the tumor tissue, with increased sensitivity and specificity. The self-assembly of this amphiphilic dendrimer with the gallium radioisotope is illustrated in Figure 23.

The first nuclear magnetic resonance dendrimer imaging study that used PAMAM G4 dendrimers as a carrier for the delivery of gadolinium ions, complexed with iminodiacetic acid, was published in 2015 [228]. Biodistribution studies have shown increased signal intensity, preponderantly in the liver, in the range of 59% to 116%, due to the conjugates between PAMAM G4 dendrimers and the complexes of gadolinium ions with iminodiacetic acid. This increase corresponds to the highest concentration of gadolinium in these conjugates, after administration.

When introducing a new contrast agent into imaging, it is important to assess the safety of its use, as well as its biocompatibility and toxicity, mostly considering its effects on homeostasis [229].

The conjugation of radioisotopes with various dendrimers reduces the dosage required to obtain images, thus improving their efficiency, mostly due to changes in their pharmacokinetics. The use of gadopentetate dimeglumine or gadobenate dimeglumine in imaging has its limitations, in particular due to their pharmacokinetics, because of the rapid transition from the blood vessels to the intestine when administered intravenously and the low contrast between the pathological tissue and the healthy one, due to its insufficient sensitivity for the recognition of pathological tissue and low contrast when high magnetic fields are applied [230].

In order to increase the relaxation rates, paramagnetic chelates are most frequently used. These are widely used in magnetic resonance imaging (MRI) as well, with the disadvantage that they are quickly removed from the blood stream. The administration of high doses of chelates to improve relaxation rates leads to an incremented toxicity of metal ions, thus suggesting that the use of dendrimeric conjugates is a promising approach in imaging [231]. Increasing dendrimer generation is an advantage for the improvement of proton relaxation, as the rate of conjugation increases with the number of terminal groups [232].

Thus, the use of PAMAM dendrimers as carriers for a contrast agent with gadolinium ions increased the relaxation time of Gd^3+^ ions in the conjugate with dendrimers in comparison to the one using a single chelated Gd^3+^ ion. Because of the increased half-life, which can reach 200 min (in comparison with 24 min for the Gd^3+^ diethylenetriaminepentaacetate (DTPA)) the dendrimer-based agents can provide exceptional contrast in MRI-examined angiograms [233]. The longitudinal relaxivity (r1) of Gd^3+^ was increased by means of second and sixth generation dendrimer chelates. As r1 is increased, the MRI signal is increased [234].

The use of dendrimeric conjugates in imaging offers distinct advantages over low molecular weight gadolinium chelates, especially its improved pharmacokinetics, which mean that much clearer images of organs (liver, kidney and lymphatic tissue) can be obtained. The development of clinical applications is increasing, especially in lymphatic imaging [235].

The development of new and improved contrast agents for computed tomography (CT) has proven to be necessary due to the lack of targeted specificity, the renal toxicity at high doses and the rapid clearing from the blood stream of the iodine-based compounds that are currently used in clinical applications. Thus, the development of contrast agents with longer blood stream circulation time, lower renal toxicity, higher contrast quality and high specificity was considered a challenge [236,237].

To improve the stability and targeting of iodine-based compounds, ^131^I-PAMAM dendrimers were developed [236]. Due to the higher X-ray attenuation coefficient than that of iodine-based compounds, bismuth sulfide and gold nanoparticles were investigated. Bismuth sulfide nanoparticles were able to attenuate the X-ray penetration more effectively than iodine-based compounds, at the same concentration of the active element, thus bismuth dendrimer-stabilized nanoparticles (DSNPs) could be used in CT imaging [237].

Gold nanoparticles were entrapped by dendrimers, such as the G5-PAMAM dendrimers that provided a good X-ray attenuation, the acetylated PAMAM dendrimers that targeted lung adenocarcinoma and increased biocompatibility, the folic acid-G2 PAMAM dendrimers that targeted mouth carcinoma and provided good cytocompatibility and a high X-ray attenuation, and the lactobionic acid-PAMAM dendrimers that targeted hepatic cancer and provided high X-ray attenuation [236].

**Figure 24:**
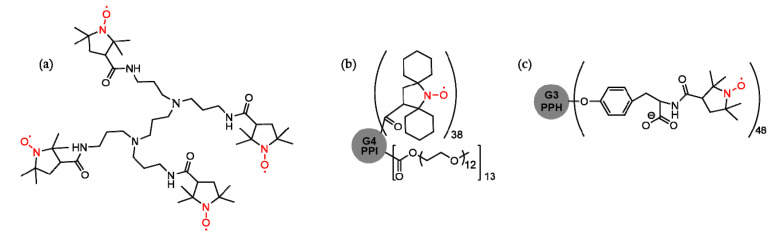
Radical dendrimers: 2,2,5,5-tetramethyl-1-pyrrolidinyl-oxy functionalized (**a**), G4 PEG-ilated spirocyclohexyl nitroxide functionalized (**b**) and G3 phosphorhydrazone with tyrosine linker, 2,2,5,5-tetramethyl-1-pyrrolidinyl-oxy functionalized (**c**). Adapted from [238,242,243], published by Magn. Reson. Med. 2002, J. Am. Chem. Soc. 2012 and ACS Appl. Bio Mater. 2020.

Radical dendrimers (like nitroxyl functionalized dendrimers—Figure 24a) were also tested as potential candidates for NMR imaging enhancers due to their paramagnetic properties. Gd^3+^ salts (Gd-DTPA) seem to have a smaller or at most equal relaxivity in comparison with relatively low generation nitroxyl dendrimers (up to three or four). On the other hand, Gd^3+^ is accumulated in the body and may exhibit toxicity risks. Radical dendrimers have low toxicity, and the main advantage that can be functionalized with many functional groups per molecule is the obtaining of an increased relaxivity. As low generation radical dendrimers are subject to rapid enzymatic decomposition into diamagnetic compounds inside the body, upon higher generation, the bioreduction rate is decreased. A disadvantage of higher generation radical dendrimers is the low solubility, but this aspect can be modulated by surface moieties [238,239,240,241].

G2 to G4 generation PPI PEG-conjugated dendrimers functionalized with spirocyclohexyl nitroxide were tested in vivo. One of them (Figure 24b) has a suitable water solubility (0.5 g/mL), enhanced relaxivity, and quite a small bioreduction rate. The signal inside the blood, the kidney medulla and the cortex allowed a long imaging time (over 90 min) [242].

G0 to G3 generation phosphorhydrazone with tyrosine linkers was functionalized with up to 48 organic nitroxide radical units (Figure 24c). These dendrimers are soluble and presented a high relaxivity (four times larger than that of Gd-DTPA), suitable half-lives (which increased together with dendrimer generation) and low aggregation at physiological pH (<0.2%), and the cell viability (when tested on a normal fetus lung tissue cell line) was almost 100% [243].

**Figure 25:**
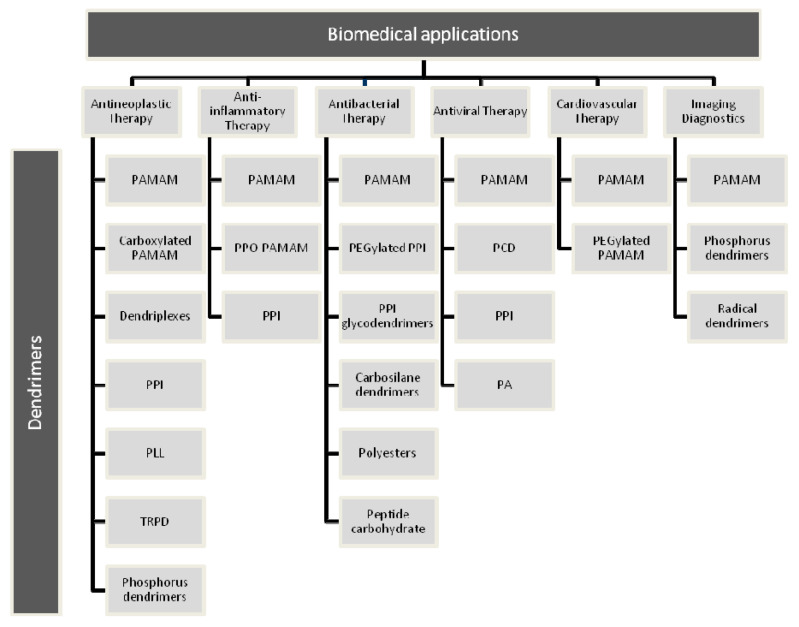
Biomedical applications for different types of dendrimers.

As presented in this paper, the types of dendrimers used for biomedical applications are summarized in Figure 25.

## 4. Toxicity Reports Regarding Dendrimers

The extended dendrimer-focused research conducted until now contributed to the characterization of these promising compounds for medical applications. Along with several major improvements that dendrimers provide, regarding pharmacokinetic and pharmacodynamics properties, toxicity elements were also reported [5,30,244].

**Figure 26:**
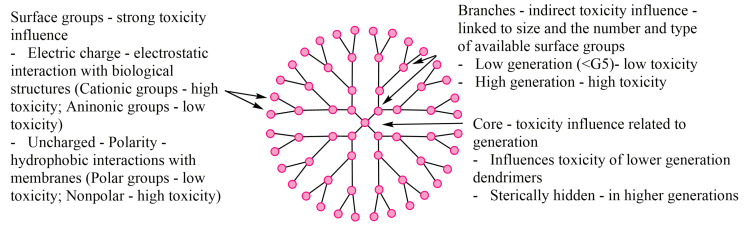
Schematic representation of the toxicity of dendrimers based on their structure [63,245,246].

Toxicity, as well as all the other properties of a compound, are directly linked to its structure. Specific elements composing a dendrimer (core, branch, surface groups) contribute to the increase or limitation of its toxicity (Figure 26).

Dendrimers can interact with biological membranes, eventually leading to a significant disruption and cell death [45]. This ability is of great toxicological interest, and strategies are under development in order to protect healthy tissue and to specifically target pathological cells [247,248,249].

A multitude of studies were conducted on the cytotoxicities of different dendrimers [26,39,40,41,42,43,44,45,46,47]. Normal and cancer cell lines of animal and human origin were studied, among which were B14 (Chinese hamster fibroblasts), N2a (mouse neuroblastoma), CHO (Chinese hamster ovary, BRL-3A (rat liver derived cells), H4IIE (rat hepatoma), HepG2 (human liver hepatocellular carcinoma), Caco-2 (colon adenocarcinoma), B16F10 (murine melanoma cells), SW480 (primary adenocarcinoma of colon), U87MG (glioblastoma), hTERT/E6/E7 (human immortalized astrocytes), HaCaT (human epidermal keratinocytes), SK-Mel-28 (human melanoma), MDA-MB-231 (breast cancer), SKOV3 (human ovarian carcinoma), HepG2 (human liver hepatocellular carcinoma) and MCF7 (human breast adenocarcinoma) [40,250].

As regards hematological and immunological toxicity, significant hemolysis was reported for PPI dendrimers G4 and G5, these possessing terminal amine groups. The toxicity was lower for galactose PPI dendrimers [251]. Furthermore, a reduction in erythrocyte number and an increase in leukocytes were reported for the PLL G4 dendrimer [252] and PPI G5 dendrimer [253]. Platelet activity interference and blood clot formation, triggered by high generation cationic PAMAM dendrimers, were reported in vitro [254]. In comparison to the previous effect, triazine dendrimers of higher generations possess a similar, milder activity. The intensity variation is considered a consequence of the terminal amine numbers [255]. The specific accumulation of dendrimers on atheromatous tissue can lead to thrombosis [256]. A cytokine response mediated by the production of reactive oxygen species was observed after macrophage exposure to PAMAM G4 to G6 dendrimers. High concentrations of these compounds can lead to cell destruction [257]. The PAMAM G5 dendrimers along PLL and polyethyleneimine dendrimers exhibited complementary activation properties [258].

As regards neurological toxicity, PAMAM G4 dendrimers increased the membrane permeability and intracellular calcium concentration of hippocampal neurons, interfering with synaptic signaling [259]. A different study showed a direct relation between PAMAM dendrimers and the oxidative stress-induced cell death of neurons [260]. PAMAM G4 dendrimers and biotinylated PAPAM dendrimers exhibit toxic effects on blood–brain barrier cell cultures [261]. PAMAM dendrimers had negative effects on the proliferation and migration of human neural progenitor cells in an in vitro experiment [262]. In vivo studies on mice revealed a neurotoxic effect via the measurement of neuronal biomarkers after the intranasal administration of a PAMAM dendrimer [263].

As regards digestive system toxicity, cationic PAMAM G4 and higher-generation dendrimers manifested a toxic effect on gastrointestinal tract models [42].

As regards hepatic and renal toxicity, mice were used for in vivo toxicity assessments. The liver accumulation of cationic G4 or higher generation dendrimers following intravenous administration may lead to hepatic toxicity [43]. High concentrations of marked, biotinylated-PAMAM dendrimers were spotted in the kidney four hours from intravenous administration [258].

Cationic dendrimers have been proven to impart the highest toxicity. In order to overcome these limitations, several approaches emerged for the surface modifications of dendrimers [31,251,264,265]. Although extended toxicity studies are still expected and necessary in order to fully characterize the safety profile of dendrimers, the fact that some products have obtained market authorizations and are available is encouraging [266].

## 5. Conclusions

Dendrimers possess many applications due to their functional and structural versatility. They can be used in different fields, like photodynamic therapy, biomedicine, the delivery of genes and siRNA, pharmacy, biopharmacy, the conjugation of oligonucleotides, immunology and imaging. As this study has shown, dendrimers are macromolecular structures with multiple advantages that can suffer modifications in order to ensure drug transport and targeted drug delivery. The toxicity of different dendrimers constitutes a limitation of their applications in biomedicine, and has triggered the development of different toxicity reduction strategies.
